# Systematic Review on Crimean–Congo Hemorrhagic Fever Enzootic Cycle and Factors Favoring Virus Transmission: Special Focus on France, an Apparently Free-Disease Area in Europe

**DOI:** 10.3389/fvets.2022.932304

**Published:** 2022-07-19

**Authors:** Célia Bernard, Philippe Holzmuller, Madiou Thierno Bah, Matthieu Bastien, Benoit Combes, Ferran Jori, Vladimir Grosbois, Laurence Vial

**Affiliations:** ^1^CIRAD, UMR ASTRE, Montpellier, France; ^2^ASTRE, Univ Montpellier, CIRAD, INRAE, Montpellier, France; ^3^French Establishment for Fighting Zoonoses (ELIZ), Malzéville, France

**Keywords:** CCHF, *Hyalomma* tick vectors, vertebrate host communities, viral transmission cycle, France

## Abstract

Crimean–Congo hemorrhagic fever (CCHF) is a viral zoonotic disease resulting in hemorrhagic syndrome in humans. Its causative agent is naturally transmitted by ticks to non-human vertebrate hosts within an enzootic sylvatic cycle. Ticks are considered biological vectors, as well as reservoirs for CCHF virus (CCHFV), as they are able to maintain the virus for several months or even years and to transmit CCHFV to other ticks. Although animals are not symptomatic, some of them can sufficiently replicate the virus, becoming a source of infection for ticks as well as humans through direct contact with contaminated body fluids. The recent emergence of CCHF in Spain indicates that tick–human interaction rates promoting virus transmission are changing and lead to the emergence of CCHF. In other European countries such as France, the presence of one of its main tick vectors and the detection of antibodies targeting CCHFV in animals, at least in Corsica and in the absence of human cases, suggest that CCHFV could be spreading silently. In this review, we study the CCHFV epidemiological cycle as hypothesized in the French local context and select the most likely parameters that may influence virus transmission among tick vectors and non-human vertebrate hosts. For this, a total of 1,035 articles dating from 1957 to 2021 were selected for data extraction. This study made it possible to identify the tick species that seem to be the best candidate vectors of CCHFV in France, but also to highlight the importance of the abundance and composition of local host communities on vectors' infection prevalence. Regarding the presumed transmission cycle involving *Hyalomma marginatum*, as it might exist in France, at least in Corsica, it is assumed that tick vectors are still weakly infected and the probability of disease emergence in humans remains low. The likelihood of factors that may modify this equilibrium is discussed.

## Introduction

Arboviruses are viruses transmitted by arthropod vectors to different hosts, which are susceptible to cause diseases threatening public and animal health (1). One of these viruses is considered as high risk of emergence in Europe is the Crimean–Congo hemorrhagic fever virus (CCHFV).

Crimean–Congo Hemorrhagic Fever was first described in 1944–1945 (2) in Russian soldiers and peasants who were exposed to ticks in Crimea and in 1956 in Congo (3). This zoonotic disease remains asymptomatic in animals while causing an acute and potentially fatal infection in humans (4). After an incubation period of 2–9 days, fever, progressive lassitude, and behavioral changes can occur, but these symptoms may be mild and go unnoticed or misdiagnosed. In the second phase, some patients (10–40%) develop intracranial and intraperitoneal bleeding, as well as a petechial rash, leading to coma or death (5, 6). Infection in humans usually occurs *via* a tick bite or from contact with contaminated body fluids of infected livestock or human patients (7). In nature, CCHFV is transmitted through an enzootic tick–non-human vertebrate–tick sylvatic cycle. Ticks, mostly of the *Hyalomma* genus, are considered biological vectors, as well as reservoirs for CCHFV, as they are able to maintain the virus for several months or even years. They are also able to transmit CCHFV from one generation to the next (vertical transmission), from one development stage to the other (transovarial transmission), from males to females during copulation (sexual transmission), or from one tick to other ticks feeding closely on a same non-viremic host (cofeeding) (8). Although vertebrate animals are not symptomatic, they can replicate the virus and be a source of infection for both ticks and humans (9), at least during viremia, which is described to last no more than 7–10 days (10). Currently, CCHF is recognized as endemic or potentially endemic in multiple areas of the world throughout Africa, Asia, and Middle East (11). In Europe and in the Mediterranean Basin, it is considered as an emerging disease, with epidemics observed in Turkey since 2002, regular cases in some countries of Balkans, and occasional autochthonous cases recently reported in Spain, as well as one case in Greece (12, 13). As CCHFV has been described in multiple tick and vertebrate species, it circulates on several continents according to the distribution of its tick vectors and different sedentary or migratory vertebrate hosts, which are amplifiers of virus and/or ticks (14). With global warming, the distribution of vectors is changing, potentially modifying CCHFV circulation and increasing the risk of virus emergence in new geographic areas (15). Indeed, the establishment of one of the CCHFV tick vectors, *Hyalomma marginatum*, was recently demonstrated in the mainland of France while it has been already reported in southern Corsica island for 50 years (16, 17). Given the human cases of CCHF recently reported in Spain and the regular exchanges of bulls, horses, and wildlife with this neighboring country, CCHFV circulation was questioned in France.

In areas potentially at risk for CCHFV introduction and circulation, serological surveillance of sensitive domestic animals is a rational system for the early detection of virus circulation (18, 19). It is even more relevant that a large panel of vertebrate animals can host *Hyalomma* ticks and CCHFV, and can develop CCHFV antibodies persisting for several years (10, 20). Sheep are known to sufficiently replicate CCHFV and to show high seroprevalence in some countries (21, 22), and are therefore considered suitable sentinels for monitoring and detecting outbreaks/circulation in new and non-endemic areas (18). Cattle are one of the common domestic hosts for the adult stage of *Hyalomma* ticks and could also be appropriate sentinels for determining at-risk areas for CCHFV circulation since they produce antibodies upon first contact with the virus (23). In France, a preliminary serological study on cattle and small ruminants was carried out in Corsica between 2014 and 2016. Seropositivity of 13% in cattle and 2–3% in small ruminants was observed and the presence of specific neutralizing antibodies was confirmed by plaque reduction neutralization test (23). This suggests that CCHFV already circulates in Corsica, although CCHFV remains to be isolated and genotyped to confirm its presence and phylogenetic origin. This study also highlighted a spatial hotspot of high seroprevalence in the northwest of the island (23). No data is currently available for mainland France but investigations are in progress to determine the epidemiological status of the continental France. Assuming that CCHFV is already circulating at least in Corsica but maybe more widely on the French territory, as any animals must be infected by CCHFV to develop an immune response, the seroprevalence measured can reflect the rate of CCHFV infection in animal vertebrates and can be a proxy for estimating the intensity of CCHFV transmission among the enzootic natural cycle.

To maximize early detection of arbovirus emergence in non-endemic areas, surveillance efforts should target areas where circulation is most likely, and thus identifying these potentially emerging hotspots is a major challenge (24). Determining ecological conditions leading to more or less transmission improves our ability to predict risks (24). Regarding CCHF, there have been several epidemiological and modeling studies examining such factors influencing virus introduction and spread, at the regional or country level (15, 25–29). However, the epidemiology of CCHF is complex and territory dependent, with host–vector–pathogen interactions that can vary from one socio–ecosystem to another (30). If CCHFV is confirmed to circulate in France (apart from ongoing serological and entomological investigation), questions arise regarding the factors responsible for its local amplification and diffusion, to explain the observed occurrence of CCHFV antibodies in ruminants without any reported human cases.

This study aims to review the literature in its entirety concerning CCHF, to revisit the CCHF epidemiological cycle as hypothesized in the French local context and select the most likely parameters that may influence the virus transmission among tick vectors and non-human vertebrate hosts. Hypotheses are also made on other possible not-yet-confirmed factors that may be specific to our French socio–ecosystem.

## Available Bibliographic Resources

**Figure 1:**
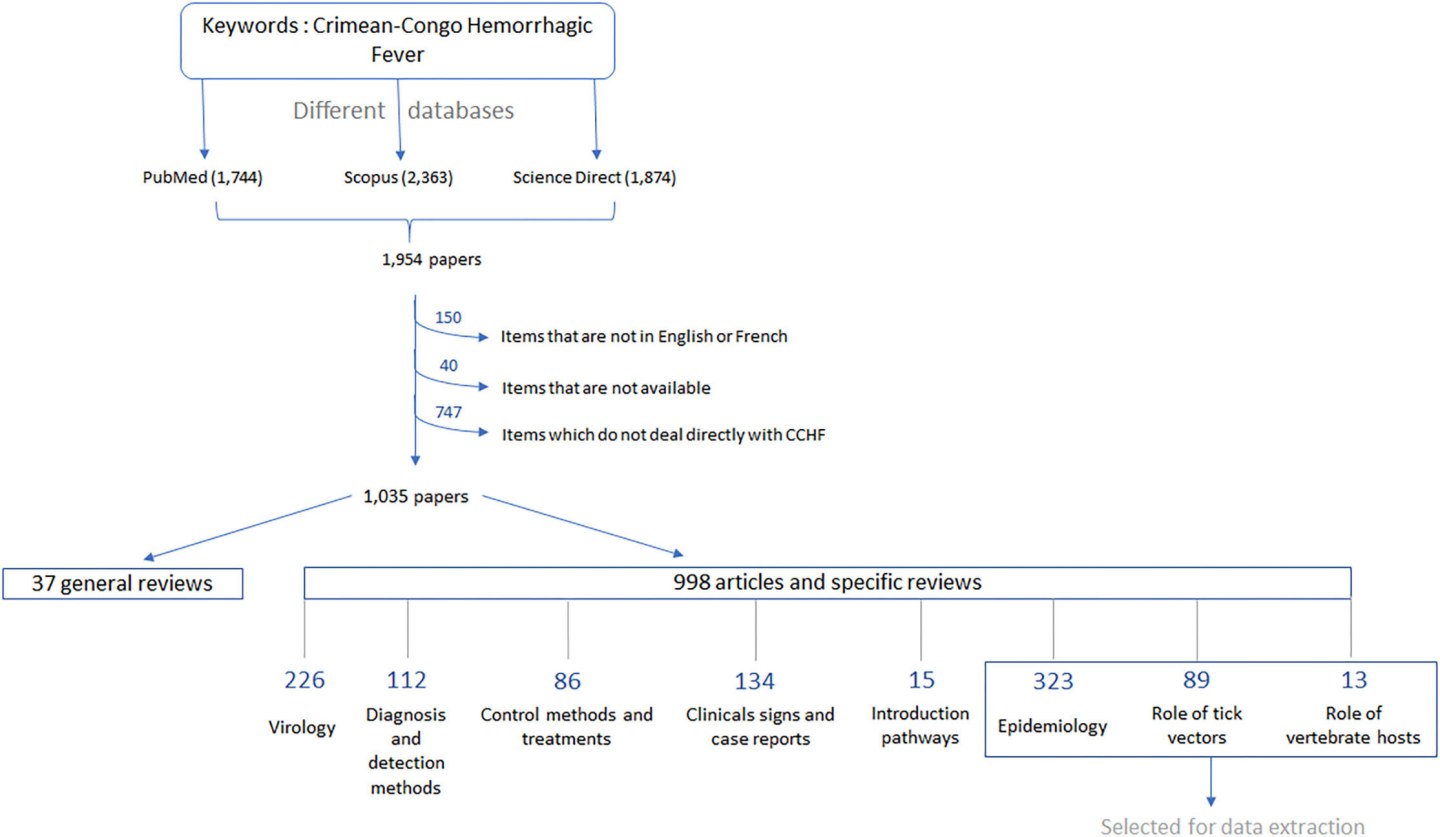
Workflow summarizing the steps taken to classify the different articles in the literature and to select the categories of interest for our study.

In 2018, Dereli and Kayser conducted an overview of scientific publications in the CCHF field, to identify new research strategies to control the virus. For our current review, we used the same search databases that produced the highest number of resources in the previous survey, namely, PubMed, Scopus and ScienceDirect, and the same keywords “Crimean–Congo Hemorrhagic Fever” under different orthographies. We framed our searches from the 1940s, the first mentions of articles dedicated to CCHF according to the previous study, to December 2021. By combining the three databases, we obtained 1994 articles (Figure 1), with an average of 123 new articles per year since 2015, the last date included in the previous study. This is in line with the reported increase of publications since the massive CCHF human outbreaks in Turkey in 2002, with an average of 100 articles per year since 2011 (31).

Bibliographic resources for which the content was neither available in French nor in English (i.e., written in Russian, Serbian, Turkish, or Japanese) were excluded, as we could not easily exploit them. We also removed articles that did not deal directly with CCHF, as no data could be extracted. Original studies, reviews, and some reports were included in this study. There was also one book dedicated to CCHF that was considered as a sum of several publications. In the end, a total of 1,035 resources, dating from 1947 to 2021, were selected for data extraction and were sorted based on their titles, abstracts, and keywords into a few categories (Figure 1). For the purpose of our review, we directly removed those related to CCHFV virology number = 226, CCHF diagnosis and detection methods (32), CCHF symptoms in the form of case reports number = 134, as well as CCHF control and treatment (33) as they were not useful for the data extraction. As we focused on the enzootic circulation of CCHFV, we also removed the articles studying introduction pathways (15). For our analysis, we kept general reviews on many aspects of CCHF (34) and those related to CCHF epidemiology number = 323, the role of ticks as CCHFV vectors (35), and the role of vertebrate animals in CCHF (13). Concerning epidemiology, we considered any studies related to the frequency and distribution of CCHFV in humans and animals, as well as the factors influencing its occurrence. This included the large number of serological surveys conducted in many different countries, serving as the basis for the first investigations on CCHF.

Publications serving specifically as information resources for data extraction were referenced as Supplementary Material. For each topic, we first considered the existing reviews as they were expected to synthetize all available data prior to their publication date. To assess their exhaustiveness and relevance, we systematically referred to part of the original cited articles, and then completed or updated data with lacking or newly published information. Regarding tick vectors and vertebrate hosts that should be considered in the CCHFV transmission cycle, we considered the historical review on the epidemiology of CCHF in Asia, Europe, and Africa published by Hoogstraal (14), as well as two specific articles, written by Turell (36) and Nalca and Whitehouse (37). More recently, Gargili et al. (8) provided an update of field and laboratory studies that have contributed to demonstrate vector competence and capacity of different tick species and genera for CCHFV. At the same time, Spengler et al. (10) proposed a systematic review of experimental CCHFV infection studies, assessing the role of many vertebrate animals in CCHFV amplification and transmission.

Some data not directly extracted from our reviewing process were also necessary to complete our understanding on tick vectors and vertebrate hosts in the French context. Concerning host preferences of potential CCHFV tick vectors, especially those of *H. marginatum*, we did not review all the available literature, as it was not the main purpose of our study. However, we made preliminary estimations based on the relative mean infestation rates and the relative proportions of ticks per host species, using what we considered to be reference articles, providing comparable and relevant data for our geographical zone of interest. For host densities in France and the capacity of hosts to spread, we used census and predictive maps available from the French Biodiversity Office (OFB), data archives from major European projects working on the prediction of vector-borne disease transmission (EDENext and Pale–Blue), as well as specific scientific articles. All the related publications or sources of these “extra” data were referenced in Supplementary Material.

## Assessment of French Tick Vectors and Reservoirs of CCHFV

Although the involvement of *Hyalomma* ticks as main vectors and reservoirs of CCHFV is confirmed and quite well-documented, the role of the other tick genera in CCHFV replication and transmission is not as clear. The epidemiological status of ticks is all the more difficult to assess, as detecting the viral genome, or even isolating the virus in a tick does not mean that the latter is able to maintain and replicate the virus, especially when collected directly on viremic animals from which it ingested the virus during a blood meal. In addition, being a competent vector for a virus (i.e., the ability to become infected during a blood meal, replicate the virus, and transmit the virus to another host through a subsequent bite) does not necessarily result in efficient transmission in nature because several other parameters, such as vector density and longevity, feeding rate, and trophic preferences of vectors, as well as the infectious period and susceptibility of vertebrate hosts, are involved in what is called vectorial capacity (36). As a consequence, replication of CCHFV in the tick throughout its development cycle but also the additional tick-to-tick transmission pathways (which characterize the ability of ticks to serve as reservoirs) are crucial in CCHF epidemiology. This is especially true when the likelihood of being infected from viremic animal hosts is low and when it is necessary for ticks to maintain CCHFV infection during periods such as winter in temperate regions such as France that limit active virus transmission (8). In addition, differential tick biology may impact their vector and reservoir roles. In Ixodid ticks, there is one larval, one nymphal, and one adult stage, which each require a bloodmeal from vertebrate hosts before molting or reproducing (38). Ticks known or suspected as CCHFV vectors can range from one-host species (where each parasitic stage feeds on the same host) to three-host species (where each parasitic stage feeds on different hosts), which impact the opportunity for a tick to become infected from a viremic animal and retransmit the virus during its lifetime (14, 39).

Tick species that have been found to be infected at least once by CCHFV and are present in France are indicated in Table 1 and related references are indicated in Supplementary Data 1. According to several criteria shown in Table 1, we were able to rank ticks according to their presupposed importance as CCHFV vectors within the enzootic transmission cycle in the French local context. We did not consider tick species that are not present in France, although they could be introduced punctually (e.g., through migratory birds for *H. rufipes* or imported ruminant for *H. anatolicum*) (16, 40), and may be responsible for CCHFV introduction, they cannot establish viable populations allowing persistent local virus transmission. Concerning *H. lusitanicum*, it apparently likely transmits CCHFV in Spain. Indeed, CCHFV has been mostly detected in such tick species compared to *H. marginatum*, and red deer on which adult stages likely engorge have been found 3 times more CCHFV seropositive than cattle in Spanish areas where CCHFV circulates locally (34). Unfortunately, there is still no experimental data to confirm its vector competence. In France, *H. lusitanicum* was historically reported in south–western part of the territory, as well as in Camargue at the east (20, 41). For the last 60 years, no new reports of *H. lusitanicum* has been referenced. Based on many field observations recorded in an extensive synthesis, Morel (42) described this species as clearly dependent on rabbit populations although Valcárcel (43) recently modulated this assertion. With the quasi-extinction of wild rabbits in France due to myxomatosis epizooties in the 1950s (44), we consequently assumed that this tick species has disappeared from the territory or at least drastically declined to remain anecdotal through residual small populations. Without any updates of presence, we thus decided not to include this tick species into Table 1, although keeping in mind its epidemiological importance elsewhere.

**Table 1:** Candidate tick species as vectors and/or reservoirs of CCHFV in France.

**Tick species**		**CCHFV isolation or RNA detection**	**Experimental evidence of vectorial competence**	**Vertebrate animal hosts**	**Number of hosts**
*Rhipicephalus bursa*		Virus isolation in Crimea, Bulgaria, Greece, Armenia, Azerbaijan, Turkmenia Antigens and RNA detection, in Greece, Armenia Albania, Kosovo, Turkey (ticks on hosts)	No data	Mostly on cattle, sheep, goat at any development stage Original hosts: roe deer, wild goat, mouflon	Three-host tick
*Hyalomma scupense*		Virus isolation in Azerbaijan, Uzbekistan, Kazakhstan, Tadzhikistan (ticks on hosts) RNA detection in Turkey, Pakistan, Iran (ticks on hosts) RNA detection in non-engorged ticks collected in the field in China	One assay where no infection in eggs hatched by infected females (data not available)	Mostly on cattle at any development stage	One-host tick
*Haemaphysalis punctata*		Virus isolation in Crimea and Moldavia	No data	Immature stage mostly on birds and hares Adults mostly on cattle, sheep, goat, and birds Reports on humans	Three-host tick
R. sanguineus sensu lato*,**	*Rh. sanguineus*	Viral isolation in Crimea and Bulgaria, RNA detection in Iran, Turkey (but not regular although other *Hyalomma* ticks are positive), antigens detected in Pakistan	Infection of salivary glands detected once by IFAT	Mostly on dogs at any development stage Reports on humans	Three-host tick
		*Rh. turanicus*	Virus isolation in Kirgizia and RNA detection in Turkey, Armenia, Greece, Iran, Bulgaria (ticks on hosts)	No data	Immature stage mostly on rodents and insectivores Adults mainly on cattle, goat, sheep	Two- and three-host tick
*Ixodes ricinus*		Virus isolation in Moldavia and Crimea RNA detection in Bulgaria, Kosovo (ticks on hosts)	No data	Immatures stage mostly on rodents and birds Adults mainly on cattle and roe deer Reports on humans	Three-host tick
*Dermacentor marginatus*		RNA detection in Turkey, Spain, Greece (ticks on hosts)	Some larvae, nymphs and adults obtained from eggs hatched by infected females were infected and able to maintain and transmit CCHFV	Immature stage mostly on insectivores, rodents and small carnivores Adults mostly on cattle, horse, sheep, wild boar, deer In Russia, concordance between D. marginatus bite and CCHF human cases	Three-host tick
*Hyalomma marginatum*		Antigens and RNA detected in Spain, Russia, Turkey, Iran, Bulgaria, Armenia, Pakistan and Kosovo (ticks on hosts) RNA detection in non-engorged ticks collected in the field in Turkey	Larvae, nymphs and adults obtained from eggs hatched by infected females were all infected and able to maintain and transmit CCHFV Very high rates of infection through infectious blood meal (up to 100%) Higher rates of transovarial transmission than other tick species	Immature stage mainly on lagomorphs and birds Adults mainly on horse, cattle, wild boar, less on sheep and goat Concordance between H. marginatum bite and CCHF human cases in Turkey	Two-host tick

*Rhipicephalus rossicus: good vector but not present in France.

**The specific identification of Rh. sanguineus vs. Rh. turanicus is difficult and thus preferred to keep the group Rh. sanguineus sensu lato.

*Colors were used to assess their role, with green, orange and red representing weak, middle, and high evidence of vector competence and/or vectorial capacity, respectively*.

As a result, and based on our bibliographic review, it appears that *H. marginatum* seems to be the best “candidate” for the transmission of CCHFV in France. It succeeded experimentally in all pathways of transmission, and is often associated with CCHF human cases in other endemic countries, and presents bioecological features favoring engorgement on potential CCHFV amplifier hosts at immature and adult stages. Its abundance in Corsica and its recent establishment in the southern mainland (16, 43, 45), in addition with the detection of CCHFV antibodies in Corsican cattle (23), make it a very suitable vector with the risk of CCHFV circulation. *Dermacentor marginatus*, which is autochthonous in the South of France (41, 46) and which adults stages can also engorge on CCHFV amplifier hosts would also be a suitable vector. Although its ability for transovarial transmission from infected females to subsequent generations was demonstrated once in the 1970s (47), no further assays have been conducted since this date and evidence of CCHFV genome detection in ticks remains scarce compared to *H. marginatum*. In the east of the Mediterranean Basin, where CCHFV circulates at high levels, several studies did not detect CCHFV in *D. marginatus*, whereas the virus was amplified from other tick species collected on the same animals (48–51). However, *D. marginatus* ticks feeding on wild boars were found to be infected in CCHF endemic areas in Spain, without determining the infectious status of animals (34). In Turkey, some specimens collected from wild boars were also found infected but this was attributed to their association with *Hyalomma* infected ticks on the same host (52). Therefore, further investigation is required to definitively confirm or deny *D. marginatus* as a CCHFV vector; however, its role as a secondary vector cannot be excluded at this stage. Apart from these two species, no other ticks present in France presented virological or bioecological criteria to be considered as potential CCHFV vectors. Historically, *Hyalomma scupense* has been described in western France and has been recently identified in Corsica (45, 53). Although it could be assumed to be a competent as any *Hyalomma* tick species, the fact that it infests the same cattle host throughout its development cycle (one-host tick) and cannot transmit CCHFV to its progeny (14) does not make this tick species a suitable vector of CCHFV. Regarding *Rh. bursa*, Gargili et al. (8) suggested that its bioecology could be favorable for CCHFV transmission but in absence of the relevant virological evidence, we did not consider this species as a suitable vector, despite its abundance in the South of France (17, 41, 54).

## Assessment of French Vertebrate Animals as Hosts of CCHFV and Tick Vectors

We considered that vertebrate animals can contribute to CCHFV transmission within the natural enzootic cycle through three major abilities: (i) by sufficiently replicating CCHFV to be able to infect tick vectors during a blood meal, (ii) by amplifying tick vector populations and providing sufficient naive ticks to be infected, and (iii) by introducing CCHFV or CCHFV-infected or uninfected tick vectors in free geographical areas through long-distance movements. Regarding the community of vertebrate hosts to be considered in a potential CCHFV enzootic cycle in the French context, we decided to focus on those infested by *H. marginatum* as it was identified to be the best current “candidate” vector to locally transmit CCHFV.

Although data are scarce (Supplementary Data 2), the monitoring of virus kinetics in experimentally infected animals is the only relevant method to assess the role of vertebrate hosts as CCHFV amplifiers. Conversely, many field investigations are based on serological tests measuring antibodies targeting CCHFV as this is very useful for early detection and provides relevant information on transmission levels (14). However, some animal species, such as horses, can develop the same levels of antibodies as any other animals although their viremia remains insufficient to allow the infection of new naive ticks to maintain transmission through blood feeding (10). As a consequence, we consider that serology likely reflects the exposure of vertebrate hosts to CCHFV-infected tick vectors, depending either on tick bite frequency or on tick infection rate. As stated above, cattle and small ruminants have often been reported as the most sensitive indicators of low-level CCHFV circulation, as they seem to be highly infested by CCHFV tick vectors (55, 56). However, this can vary according to the geographical region and the local CCHFV transmission cycle, and does not predict their ability as CCHFV amplifiers. Another limiting aspect of using field serological surveys is the difficulty to compare results, as many different techniques with distinct sensitivity and specificity were used over the years and between studies (55). As viremia is short in any animal, reports of direct CCHFV detection in field animal samples remain too scarce to be used to assess their relative role in CCHFV replication. The few symptoms in animals, when present, are not informative on their role as CCHFV amplifiers since they do not seem to be correlated with viremia (10).

Regarding the ability of vertebrate animals to amplify tick vector populations, this mainly depends on host preferences and abundance of tick vectors, but also on the availability of vertebrate hosts for ticks. Theories are very extensive on host preferences in ticks, ranging from the necessity for ticks to be specialist and co-evolve with their hosts, and consequently their environment (57), to their likely adaptation to abiotic conditions and specific habitats resulting in host preferences (58). This question remains difficult to assess, as experimental studies providing practical measures are few to none. For *H. marginatum*, only one study tested the efficiency of its development cycle according to its blood feeding on three different small vertebrates (mice, guinea pig, and white rabbit), which led to a laboratory animal model useful for CCHFV transmission experiments (59). Two field surveys were also conducted to test the effect of host species and host abundances on population densities of *H. aegyptium* and *H. lusitanicum* (60, 61). For the latter, it was shown that the experimental removal of hares from the environment was significantly correlated to a lower abundance of *H. lusitanicum*, although it did not prevent its presence. Based on such partial results, field data reporting infestation rates of several host species by *H. marginatum* or relative proportions of *H. marginatum* among all tick species found on different hosts may be a proxy to assess host preferences of this tick species. These studies are numerous, at least for common domestic hosts such as cattle, small ruminants, and horses (Supplementary Data 2). However, scientists have usually investigated ticks from livestock because of its economic importance and the confirmed side effects of ticks and tick-borne infections on productivity. The same phenomenon can be observed on emblematic wild vertebrates and this may bias comparisons between vertebrate hosts. For *H. marginatum*, only one study is available on its presence on a striped field mouse (*Apodemus agrarius*) in Bulgaria (62). However, because rodents are frequently examined for ticks as they are reservoirs for Lyme Borreliosis and ick-borne encephalitis causative agents, we can assume that *H. marginatum* never, or extremely rarely, parasitizes rodents, as suggested by Hoogstraal (14). Another limitation of using field data on tick collections from hosts to assess host preferences is the apparent heterogeneity and incongruences between geographical regions, as it is observed for example for cattle and small ruminants in Romania compared to Turkey, and even within Turkey (52, 63, 64). This could be due to either distinct abilities of ecoclimatic zones to host *H. marginatum*, which may result in various tick densities, or distinct host communities available for this “generalist” tick. This last case suggests that some ticks could adapt their host preferences according to local contexts and thus we should remain very cautious when interpreting tick data collection from the field.

Finally, to assess the role of hosts as spreaders of CCHFV or CCHFV tick vectors, the most important information is their capacity to move long distances, as well as the duration of tick attachment on the host for blood feeding. As many vertebrate animals show short CCHFV viremia, the probability of movement during viremia is very low; they are therefore more likely to spread ticks infected with CCHFV than to directly spread the virus *via* physical contacts with contaminated fluids. Hard ticks at all development stages, remain attached to their host during blood feeding for ~10 days, until they become completely engorged (65). However, this duration may increase if the tick species in question reduces its free-living phases, like *H. marginatum* for which larval and nymphal stages engorge on the same small vertebrate for an average of 14–21 days without falling to the ground for larval molting (20, 59, 66). Although some authors do not support this assumption (10), this period largely covers the duration of Trans-Mediterranean bird migrations, known as major hosts for immature stages (67, 68). Additionally, the commercial trade of livestock may result in long-distance movements of ruminants, potentially infested by CCHFV-infected tick vectors, as their external parasitic status is not necessarily controlled for importation (69). While domestic animal trade is common between European countries, inter alia, from Spain where CCHFV is known to circulate among domestic ungulates, importation of livestock from non-European CCHF-endemic areas into Europe remains rare (70). Occasional wildlife trans locations, such as the reintroduction of the Spanish ibex (*Capra hircus*) into the transboundary Pyrenees mountains, remain infrequent and highly monitored; however, this can be considered a practice at-risk since CCHFV antibodies have been detected in Spanish Ibex (71). In addition, the international trade of game species for the hunting industry can represent a potential source of introduction into new territories (72). Wild rabbit and hare imports have been reported from Spain since the 1970s to restock French hunting reserves and palliate the disappearance of native species due to myxomatosis and rabbit hemorrhagic disease (73, 74).

Based on the different criteria discussed above, Tables 2A,B, presents an assessment of the different vertebrate hosts of *H. marginatum* for their role in CCHFV circulation within the natural enzootic transmission cycle (reference articles are indicated in Supplementary Data 2). Voluntarily, we did not include exotic species, present in zoos or other public reception structures as their presence remains sufficiently rare to not significantly change transmission levels. This is the case for camels that are assumed to be good CCHFV amplifiers and can be parasitized by *Hyalomma* ticks (75), ostriches (*Struthio camelus*), African starling (*Lamprotornis* sp.), and red-beaked hornbills (*Tockus erythrorynchus*), the latter three representing the only bird species able to develop sufficient CCHFV viremia to infect ticks (76, 77). In the case of ostriches, they were also able to contaminate humans by direct contact in African slaughterhouses (78). However, such animals are not naturally and endemically found in France.

**Table 2:** Candidate vertebrate hosts of *H. marginatum* and CCHFV that are present in France.

	**Species**	**CCHFV amplifier (source of infection for ticks)**	**Tick population enhancer (key host to obtain a viable tick population)**	**Tick carrier (source of ticks infected or not)**
**(A)**				
Host of immatures	Hedgehog	Successful infection by inoculation, viremia (2–6 dpi) (4log), successful infection of ticks only for Hemiechinus auritus Not for *Erinaceus europaeus* that is present in France	Infestation (Hungary, Ukraine, Turkey, ex-USSR): IR: one report only (0.4%) RP: average of 4–5% [0.02–9] Density: From 4.4/km^2^ in rural areas to 36.5/km^2^ in urban areas in France	Short-distance movements (0.5–3 km; home range of about 2 ha, up to 50 ha for some males)
	Lagomorphs	Hare (*Lepus saxatalis and L. europaeus*) Successful infection by tick bite and inoculation, high viremia (1–15 dpi) (4–5log), successful infection of ticks, no symptom In Turkey, CCHFV genome detected in 7% of hares Rabbit (*Oryctolagus cuniculus*) Successful infection by inoculation, sufficient viremia to infect ticks, no symptom	Hare Infestation (Spain, Italy, Ukraine, Turkey): IR: 5–14% to 100% RP: 57–100% (according to season and zone) In Crimea and Balkans, hares highly infested but less than rooks Density: 15–20/km^2^ (south of France) Rabbit Infestation (Portugal): IR:2%, RP:1% Density: 0,01/km^2^ in France	Short-distance movements Hare: variable home range (20–140 ha) Rabbit: low home range (<10 ha) Imports of hares and rabbits from Spain
	Rodents	Only African rodents tested, which are not present in France In some species: successful infection through inoculation but too low viremia (1–4log) to infect ticks	Infestation of rodents and shrews is the exception, contrary to other tick species (ex-USSR) During the favorable season, only one report of a tick found on *A. agrarius* (Bulgaria) Variable according to species but high densities	Short-distance movements Variable according to species
	Birds	Majority of species develop no viremia (refractory): experimental infections failed on fowls, doves, and rooks. African partridge (*Numida meleagris*): successful infection through inoculation but viremia not easily detectable to assess their ability to infect ticks	Infestation (numerous reports in southern and northern Europe): IR is always low apart for some bird families that are ground-feeding (Muscicapidae, Turdidae-like blackbird, Corvidae like rook and magpie, Phasianidae like partridges, Strigidae like owls) (30–100%) Within these species, RP always high if favorable season Variable densities according to species For partridges, farms do not settle in areas where *H. marginatum* is present	Long-distance natural migrations for some species, different routes (trans-Sahara, intra-EU)
**(B)**				
Hosts of adults	Cattle	Successful infection through inoculation and tick bite, high viremia (4–6log) (2–8 dpi) sufficient to infect ticks, no symptoms except appetite loss and lethargy in some calves	Infestation: RP variable according to zone (5–10% to 73%) (south of France 22%) Low density in south of France (except Camargue)	Long-distance movements through trade (from Spain) and national transhumance
	Sheep	Successful infection through inoculation and tick bite, high viremia (4-6log) (2-10 dpi) sufficient to infect ticks, even on pre-immunized non-viremic sheep Mild symptoms (fever, liver and kidney dysfunctioning, abnormal cell count), antibodies transmitted to lamb during at least 2 months	Infestation: RP variable according to zone (0.03–0.05% to 82%) (south of France 4.4%) Variable densities, some intensive production in south-western France	Few importations and national transhumance
	Goat	No experimental data available	Infestation: RP variable according to zone (0% in south of France) Low density everywhere in France	Few importations
	Horse	Successful infection through inoculation, too low viremia to infect ticks, mild symptoms (fever, lethargy, inflammatory syndrome)	Infestation: High RP (42–78% at favorable season) Relatively high densities in France, especially Camargue	Long-distance movements through trade (from Spain and Italy), national transhumance equestrian competitions
	Donkey	Successful infection through inoculation, too low viremia to infect ticks, no symptom	Anecdotal infestation Low densities in France	Short-distance movements
	Wild boar	No experimental data available In Turkey, CCHFV genome detected in 8% wild boar tested In Spain, D. marginatus collected on wild boars are CCHFV positive	Infestation remains low (Italy, Spain, Portugal, France): IR: 2–8% and RP: 3–5% (except 69% in a report of Spain and 29% in Turkey) Very high densities in south of France (difficult to estimate)	Short-distance movements (<5–10 km; home range of 300–500 ha; up to 3,000 ha for males) Few and only illegal importations since 2018
	Roe deer	No experimental data available	Only one proof of infestation in Israel (RP: 13%) Low densities in scrublands from south of France where *H. marginatum* is abundant	Short-distance movements (home range from 35 to 150 ha)
	Red deer	No experimental data available In Spain, supposed to be good CCHFV amplifier as human cases occurred where red deer and H. lusitanicum are present. Red deer shows higher CCHFV seroprevalence (70%) than cattle (16%) in areas infested by infected Hyalomma ticks	Low infestation compared to *H. lusitanicum* that is the main ectoparasite of red deer in Spain (no information elsewhere) Low densities in France, concentrated in humid forest where *H. marginatum* is absent	Short-distance movements (home range from 800 to 3,000 ha)

*IR, infestation rate of hosts by H. marginatum (number of hosts infested among hosts examined); RP, relative proportion of H. marginatum among other tick species infesting the same host species (number of H. marginatum among the whole amount of ticks infesting one-host species).*

*Their role as amplifiers of CCHFV, amplifiers of tick populations, and carriers of ticks infected or not with CCHFV is informed, with colors from green, orange to red for low, middle, and high evidence, respectively. A, Host of immature stages; B, Host of adult stages*.

Considering Tables 2A,B, lagomorphs, especially hares, seem to play a major role as CCHFV amplifiers, as well as being a preferred host with birds for immature stages of *H. marginatum*. According to Hoogstraal (14), hares would be the vertebrate hosts mainly involved in the circulation of CCHFV in endemic areas. French populations of hares are stable but very heterogeneous depending on the habitat (74, 79), while wild rabbits remain rare despite repopulation actions. Likewise, some families of birds, especially those feeding on the ground and that can be easily infested by hunting ticks such as *H. marginatum*, are reported as important hosts for immature stages. In Cyprus and Spain, resident blackbirds and rooks showed very high infestation rates (80, 81). Their role as tick population amplifiers is all the more reason to consider these birds, as they are largely distributed in many diverse habitats of France. In addition, some representatives of these families are migrating birds connecting African and European CCHF endemic areas to France and their ability for spreading ticks potentially infected by CCHFV should be pointed out. Indeed, the recent tick collections conducted in the South of France have reported the presence of immature stages of *H. marginatum*, but also *H. rufipes* that is only established in Africa, from migrating Eurasian Blackcaps (*Sylvia atricapilla*), dunnocks (*Prunella modularis*), and European robins (*Erithacus rubecula*) (16). However, most birds are considered refractory to CCHFV and may not act as CCHFV sources (10). Considering adult stages of *H. marginatum*, the main hosts are domestic ungulates, especially horses and to a lesser extent cattle. Horses are frequent in the South of France whereas cattle farming remains rare, except in Corsica where *H. marginatum* is highly abundant (17) and in humid pastures of Camargue for bull rearing where *H. marginatum* cannot develop (82). Both species may be involved in long-distance tick spreading due to international trade, national seasonal transhumance, or even equestrian competitions. However, only cattle have been reported as good CCHFV amplifiers whereas horses cannot be sufficiently viremic to infect ticks (10). Sheep were also demonstrated to efficiently replicate CCHFV but they are very rarely infested by *H. marginatum*, at least in France (17). The role of wild ungulates remains partially unknown and deserves further investigation, although red deer (*Cervus elaphus*) has been reported to be an important host for the Spanish tick vector *H. lusitanicum* and are suspected to be efficient CCHFV amplifiers (34). However, this tick species is apparently absent from France and red deer populations are not abundant in the French area colonized by *H. marginatum* and seem to be exceptionally infested by this tick (34).

## The Presupposed CCHFV Transmission Cycle in France: Importance of Tick Vectors and Vertebrate Host Communities

Few articles describe the entire CCHFV transmission cycle (Supplementary Data 3). Those that exist are mostly dealing with mathematic modeling used to describe generic transmission cycles or surveys that focus on disease epidemiology in some endemic areas, mainly eastern ones. Other publications addressing CCHFV transmission pathways usually focus on one aspect of the viral cycle, such as the role of tick vectors, the status of vertebrate hosts or potential routes for virus transmission. Among the proposed cycles, generic ones that are deliberately simplified to model reality, cannot reflect the total diversity of tick species and vertebrate animals interacting in CCHFV transmission. For example, in West African Sahelian regions, *H. truncatum* has been considered to be the main vector of CCHFV for humans. However, some “helpers” may locally favor virus transmission and long-term persistence (39). As only 17% of CCHFV transovarial transmission and <1% of CCFV cofeeding is reported in *H. truncatum* (83), adult ticks may likely become infected at the immature stage through feeding on viremic lagomorphs. However, such small vertebrates are scarce in the region and cannot allow frequent infection in ticks. Fortunately, another tick *H. rufipes*, present at similar latitudes, was demonstrated as a possible vector of CCHFV, at least within the enzootic cycle; thanks to a large infestation of immature stages on the abundant red-beaked hornbill, the only bird able to amplify CCHFV and replace “traditional” lagomorph amplifiers (76). In southern Sudanese regions where *Hyalomma* ticks are scarce, *Amblyomma variegatum*, another tick with a large diversity of vertebrate hosts was also demonstrated to participate in CCHFV transmission. This tick species has a diversity of vertebrate hosts, including “traditional” and abundant cattle amplifiers, parasitized by each developmental stage of this tick species. Its anthropophilic behavior may also make it a good candidate for CCHFV transmission to humans (39). The above example clearly shows the risk of biased predictions when oversimplifying such complex virus epidemiological cycles or adapting previously described cycles to another geographical area, as tick and vertebrate host communities may differ and change the dynamics and levels of virus transmission. Regarding the recent emergence of CCHF in Spain, one could not have predicted that the virus would likely circulate within red deer living in forested areas and that the tick vector would be *H. lusitanicum*, rather than *H. marginatum*, the latter being more widely distributed within the country and commonly considered as the main CCHFV vector in southern Europe and northern Mediterranean countries (34, 84).

**Figure 2:**
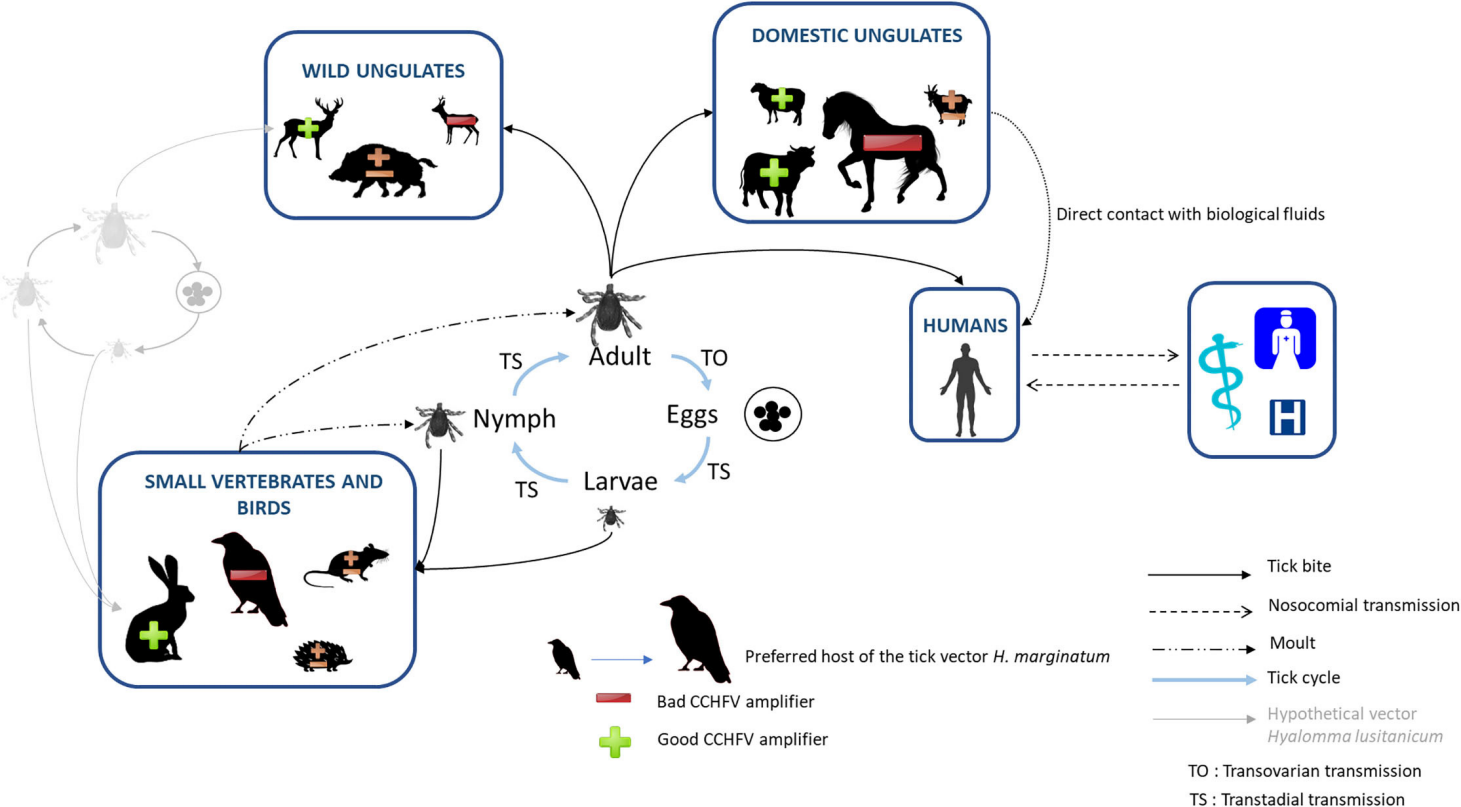
Presumed enzootic transmission cycle of CCHFV in France, involving the candidate tick vector *H. marginatum* and its different vertebrate hosts. Animals are represented in different sizes according to relative host preferences of *H. marginatum*. The ability of these animals to replicate CCHFV is indicated by “+” for good CCHFV amplifiers and “–” for bad CCHFV amplifiers.

Considering the assessment of ticks and vertebrate hosts in CCHFV transmission detailed above, the pre-supposed enzootic CCHFV transmission cycle in France can be mapped as illustrated in Figure 2. In our state of knowledge on vector competence and French tick distribution, *H. marginatum* is considered the only “serious” local candidate for CCHFV transmission. As we hypothesized that *H. lusitanicum* may be absent or only present in residual populations on our territory, it seems unable to maintain a sustainable and perennial transmission of CCHFV. In the South of France, immature stages of *H. marginatum* may likely engorge on birds, which are much more numerous in the environment than lagomorphs, though both animal classes are considered preferred hosts. However, as birds are refractory to CCHFV, the probability for ticks to become infected at immature stages and the resulting ability of emerging adults to further infect large ungulates through biting are low. This might be however modulated if cofeeding on birds may occur, but transmission rates using this pathway seem to remain low (85) and we have no specific data for *H. marginatum*. Moreover, adults of *H. marginatum* mainly parasitize horses, which are both the preferred hosts and also abundant in the south of France, but cannot develop sufficient viremia to infect ticks. Except if cofeeding may also occur on horses, their role for CCHFV transmission seems to be low to null. Blood feeding on confirmed good CCHFV amplifiers, such as cattle or sheep, remains infrequent or rare and therefore cannot strongly participate in increasing infection level in the tick vector. Regarding wild ungulates, as stated above, only red deer was assumed as good CCHFV amplifiers, although they remain quasi-absent from southern French areas where *H. marginatum* is established, and are considered unlikely hosts for this tick. Without any more information about the ability of other wild ungulates, especially wild boar, to participate in CCHFV replication or in the amplification of *Hyalomma* tick populations, their role cannot yet be fully considered. In the current state of our knowledge and our assumptions on CCHFV circulation in France, opportunities for *H. marginatum* ticks to become infected and to infect new animals would be rare, at any development stage, even if transovarial transmission and cofeeding are partially efficient. Therefore, the risk for humans to be contaminated through either tick bite or direct contact with animals' body fluids can currently be estimated as low. This prediction is based on the theoretical concept of the “dilution effect,” developed by LoGiudice et al. (86) for Lyme disease and emphasizes the impact of host biodiversity and community composition of ecosystems on their functions. The authors identified “dilution hosts” as animals characterized by high tick burden, low reservoir competence, and high population density, as could be the case of horses and/or birds in the French context of CCHFV transmission. Therefore when the host community is less diverse and mostly composed by “dilution hosts,” a “generalist” tick such as *H. marginatum* is likely to engorge on these hosts and show low infection prevalence (86). This could partially explain the detection of antibodies targeting CCHFV in French domestic ungulates, yet the lack of CCHFV detection in tick and the lack of CCHF human case reports, for example. However, we cannot omit other possible explanations, such as the existence of low virulent CCHFV strains resulting in particularly few (or no) hemorrhagic symptoms, or CCHFV-like viral strains that may be able to cause immune cross-reaction in domestic ungulates but would be too divergent to be detected in ticks using specific CCHFV molecular amplification methods. In addition, although we estimate the risk of CCHF emergence in France as low considering its presumed local transmission cycle between animals and ticks, this situation is not static and is likely to change under climate and global changes, as observed in Spain (69).

## Factors That May Promote CCHFV Enzootic Transmission in France: Intrinsic Sensitivity and Modulation of Exposure

**Figure 3:**
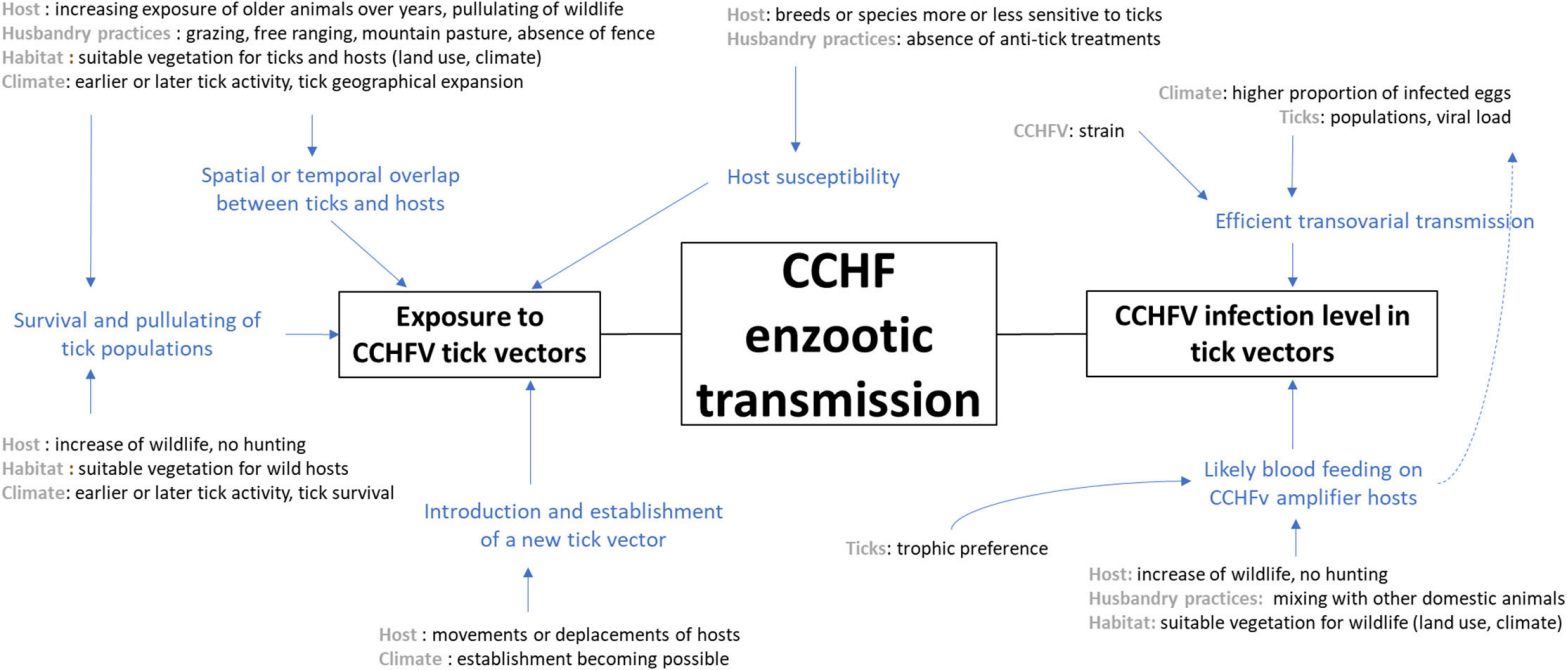
Scheme representing factors that may impact either the exposure of animals to tick vectors or the CCHFV infection level of tick vectors, which could finally influence the CCHFV enzootic transmission in France.

In the literature, some articles do refer to risk factors involved in CCHFV transmission. Among the 58 articles referenced in our review as epidemiological studies investigating risk factors for the transmission of CCHFV, 34 looked at factors favoring human contamination (usually measured by the incidence of disease cases or the seroprevalence of CCHFV antibodies in humans) and 24 referred to biotic or abiotic factors impacting the natural circulation of CCHFV among vertebrate animals. Based on the latter publications, we were able to schematize the effects of the major factors cited, either on the exposure of vertebrate hosts to CCHFV tick vectors or the prevalence of CCHFV infection among tick vectors. We determined which factors have proximal or ultimate effects and interact together, and we only considered factors in Figure 3 that could make sense within the local French context of CCHFV enzootic transmission.

Several studies conducted in Africa and Asia have shown that CCHF seroprevalence significantly increases with the animal age, either in cattle, small ruminants, and camels (33, 35, 87–97). This correlation was mainly attributed to an increasing probability of an animal to be exposed to infected tick vectors, contract CCHFV, and develop an immune response in relation with increasing age (33). Such an additive effect of age seems obvious as antibodies are assumed to persist for a long period in animals, up to several years (98). An additional potential age-linked relevant factor for exposure to CCHFV is variation in tick burden associated with body size, physiological age-linked variations, e.g., immune competence, and behavioral patterns (99). However, other parameters may explain such patterns, such as diverse breeding practices. In Sudan, when assessing age as a risk factor for cattle CCHFV seroprevalence, the authors showed that calves started to get infected after the age of 2 years, as this was when animals were released to pasture and therefore became exposed to infected ticks and subsequently to CCHFV infection (35, 89). In Mauritania, this hypothesis of overexposure in older animals has been also proposed to explain an apparent higher seroprevalence in camels than in cattle or small ruminants, as camels are bred for longer periods of time (95). Although we agree that the age effect may be a confounding factor with animal species, most ticks present host preferences that could result in differential infestation rates according to the vertebrate species. In addition, tick vectors and host species may be not homogeneously distributed, with some vertebrates being over-represented locally and therefore more infested by the tick vectors in question, present in the same zone. The investigation of both age and species effects in a serological survey conducted on small ruminants from Pakistan confirmed significant differences of CCHFV seroprevalence between goat and sheep that were not due to age, as both species are typically kept no more than a few years before slaughtering (88).

Regarding other intrinsic host-related risk factors, significant differences in CCHFV seroprevalence were also detected on several occasions depending on the breed of the animals. In Sudanese cattle, the highest rates of infection have been observed in cross breeds, compared to endogenous ones, as they seemed to be very susceptible to tick infestation (89). Relative resistance of native breeds to tick infestation has often been described in relation to distinct patterns of transmission for several tick-borne pathogens (100–102). However, concerning CCHFV, significant differences were not systematically highlighted, even within the same country (35), and in some cases the tendency was just the opposite (88). The effect of breeds was also reported in camels from Sudan but each breed was actually representative of one locality of the country and may likely reflect local tick exposure (90). In cattle, differences in husbandry practices may also occur depending on the breed, as these breeds do not show the same aptitudes for production. Production systems have their own organizational constraints. Consequently, practices related to animal movements can modify the exposure of animals to CCHFV tick vectors, as discussed in the following. This aspect should be investigated especially in French areas, like Corsica, where traditional cattle breeds are maintained and, suckling farming with free-ranging has become popular and widespread. Moreover, parasite resistance may be influenced by cross-breeding—between the Corsican breed and Limousine, Aubrac, or Charolaise—that some farmers do to increase their production compared to the Corsican breed adapted to harsh scrublands and supposed to be more resistant to ticks.

Apart from the breed, sex as another host-related risk factor was also tested in several studies and its effect was mostly insignificant on the CCHFV seroprevalence (88, 91, 96, 97). The only exception was for cattle from Malawi and South Africa where CCHFV seroprevalence was higher in females than males, although this cannot be attributed to intrinsic resistance in females against ticks (33, 87). In these countries, female cattle are raised mainly for breeding purposes, meaning they spend more time in the fields grazing and therefore have an increased risk of tick exposure. Males, however, are used for drought power and stay away from pastures longer than females and young cattle, particularly during the rainy season when crop cultivation is at its peak and ticks are active (33). In two studies conducted on cattle from Sudan, animal body condition was also tested in relation to CCHFV seroprevalence but its effect was insignificant to the assumption that poor body conditions may invoke higher susceptibility of CCHFV infection in animals (35, 89). Conversely, the possibility that CCHFV infection induces a poorer body condition in infected animal is considered anecdotal, since no clinical signs have been detected in animals to date.

As stated above, husbandry practices, such as feeding systems, can be important factors impacting the exposure of animals to tick vectors and thus there exists the risk for CCHFV transmission. A few studies conducted in Africa and Asia reported higher CCHF seroprevalence in animals grazing in pastures than those fed on trough (87, 88), and even higher prevalence in nomadic herds covering long distances (89). In Iran, Lotfollahzadeh et al. (93) also pointed out vegetation where grazing took place as an additional factor that may favor the presence and abundance of ticks and thus exposure to CCHFV vectors. In the South of France, extensive farming systems where animals are highly exposed to ticks do exist, as observed for cattle rearing in Camargue. However, these areas are of wet habitats and have been demonstrated as unsuitable for the establishment of the *H. marginatum* tick vector (103). Extensive farming can also be temporary as mostly reported in Corsica where most cattle are free-ranging in suitable scrubs during spring and summer, corresponding to the activity period of the adult stages of *H. marginatum*. Another husbandry practice that was reported as an important factor modifying the risk of CCHFV transmission is the use of acaricide treatments on animals, as they decrease the infestation load and the susceptibility of animals to ticks and subsequently the probability of CCHFV transmission. This phenomenon was reported in cattle from Pakistan (88) and was considered a reliable preventive strategy when animals from Sudan were introduced into Saudi Arabia (104). This was clearly linked to the tick burden of animals, which was also measured in some studies (33, 35, 87–91). Conversely, some studies reported insignificant or opposite effects of acaricide treatments or tick control actions but a little information was provided on the chemicals and methods used to assess their reliability (35, 87, 89). Although tick infestation rates may be lower in France than those observed in tropical or subtropical regions, some farmers use acaricides or insecticides, punctually, the latter targeting flies, or anthelmintics with ivermectine that can be indirectly active on ticks. These practices should be tested as factors influencing the exposure of animals to CCHFV tick vectors. Finally, several other husbandry practices, such as the flock size, the import of animals into farms, the slaughtering of animals on farms, or the contact with other farms, have been tested in some studies but remained insignificant (87, 88). Two factors captured our attention as they seemed relevant within the French context and could impact not only the exposure of animals to tick vectors but also the level of infection in tick vectors are as follows: (1) The presence of other domestic animals on farms and (2) the possible contact with wildlife (87, 88). As stated above regarding the importance of the host community composition for tick vectors, changes may occur in CCHFV prevalence among tick vectors depending on the probability of these ticks to feed on other viremic and infectious animals reared in conjunction with cattle. In the South of France where *H. marginatum* has been established, sheep farming and the use of horses for cultivation were previously considered traditional practices. However, farmers have progressively diversified their activities since the 1960s by including suckling cattle for meat production and dairy goats; all these animal species presenting differential abilities to replicate CCHFV and amplify tick populations. Similarly, spatial overlap between cattle and wildlife, such as lagomorphs, birds, or wild boars in absence of fences or due to free-range farming, as observed in Corsica, may change the probability for tick vectors to become infected with CCHFV.

However, apart from the husbandry practices, contact between cattle and wildlife can also result from changes in land use (21). For example, CCHFV emergence in Turkey since 2002 has been mainly attributed to the local increase of hares' abundance, which highly amplified populations of *H. marginatum* and enhanced their infection with CCHFV. This was mainly due to security issues resulting in the temporary prohibition of hunting and agricultural activities, followed by the reopening of these areas after several years with the sudden exposure of humans to highly infectious ticks (105). This was also concomitant to higher spring temperatures over several successive years within the country that accelerated the development cycle of the tick vector *H. marginatum*, decreased its density-independent mortality, and thus increased populations available to become infected (106). Kulichenko in the Russian Federation (106) and Vescio in Bulgaria (107) also mentioned climate as a main factor impacting the development of CCHFV tick vectors and thus local virus circulation. Climate can also increase tick search activity and host parasitic load, which promotes virus circulation (24). In a deterministic model considering the total development cycle of *H. marginatum* and each CCHFV transmission pathway between ticks and vertebrate hosts, Estrada-Pena and collaborators (108) showed that increased temperature, especially in late summer, allows for increased oviposition events in females before unsuitable autumn conditions, which increases survival probability of developing eggs. However, he also demonstrated that a key factor for CCHFV transmission by *H. marginatum* was a climate-independent biological parameter intrinsic to the tick vector, namely the rate of CCHFV transovarial transmission (108). Although few studies described the vector competence of *H. marginatum* for CCHFV, including tick-to-tick transmission processes such as transovarial transmission, it is assumed that this ability depends on both the tick population/species and the viral strain, as it has been well-described for other arboviruses (109, 110). Nevertheless, if CCHFV transovarial transmission is efficient, better egg survival due to higher temperature is predicted to result in a higher proportion of infected eggs. Consequently, it is possible to assert that climate change may impact both the exposure of animals to tick populations and also the level of CCHFV infection in the tick vectors. Climate change is also likely to expand the geographical range of *H. marginatum* northward in the Mediterranean Basin, with ticks recently being found in southwestern Europe (32, 111), as well as contribute to concomitant geographical spread of CCHFV from neighboring endemic areas. Indeed, *Hyalomma* ticks prefer warm summers, relatively mild winters, and reduced precipitation, which is becoming normal under the current climate change in this region (21, 112). Successful establishment of new CCHFV tick vectors in France such as *H. lusitanicum* or *H. rufipes*, due to the combined climate change and introduction events, may also occur. In relation to global warming, habitats can also change and become more or less suitable for both the survival of CCHFV tick vectors and the increased abundance of some wild mammal species, such as wild boars (113). In southern Europe, including the South of France, specific categories of xerophilous land covers such as shrubs, grasslands, and herbaceous vegetation may expand under climate change and favor the establishment of *H. marginatum* (103) among locally abundant small vertebrates population such as hares and rabbits (74). In addition, modifications in land use and husbandry practices, as a consequence of either climate or global changes (e.g., land conversion to pastures through deforestation), can contribute to an increase of open areas. Inversely, habitat reforestation due to agriculture decline can contribute to a higher abundance of wild ungulates populations, while fragmented habitats with wooded areas can increase their movement and the transportation of adult ticks to the new environments (30, 107). It appears that the environmental conditions are the ultimate factors that impact all the pre-cited factors.

## Conclusion

France seems to be still an apparently free-disease area for CCHF, as there have never been any reports of human autochthonous cases and the virus has never been detected in ticks. However, CCHFV antibodies have been detected in domestic ruminants in Corsica Island, suggesting local transmission of the virus at least in this part of France. In this work, we have shown that *H. marginatum* seems to be the best candidate for this transmission, as its distribution has been increasing in the south of France, for some years, under climate changes. The known vertebrate hosts of this tick are present and numerous locally, which also enhanced its establishment. Some of these hosts such as lagomorphs or cattle are considered as good CCHFV amplifiers while others like birds or horses are not. Although the circulation of the virus seems to be possible as shown by serological evidence in cattle, the trophic preference of *H. marginatum* and the availability of hosts may impact the probability for *H. marginatum* tick vectors to become infected though what is called “the dilution effect” and therefore to efficiently transmit CCHFV. The natural enzootic transmission of CCHFV between ticks and non-human vertebrate hosts, commonly measured by seroprevalence in animals, depends on either the exposure of such animals to tick vectors or the level of infection of such ticks. These two parameters can be impacted by various factors related to the hosts, husbandry practices, habitat, and climate.

## Data Availability Statement

The original contributions presented in the study are included in the article/Supplementary Material, further inquiries can be directed to the corresponding author/s.

## Author Contributions

CB: bibliographic review, formal analysis, writing the original draft, and reviewing and editing the manuscript. LV: bibliographic review, formal analysis, conceptualization, reviewing and editing the manuscript, supervision, and funding acquisition. PH and VG: conceptualization and reviewing and editing the manuscript. MTB: bibliographic review and review and editing of the manuscript. FJ: scientific support and review and editing. MB and BC: funding acquisition and reviewing and editing the manuscript. All authors contributed to the article and approved the submitted version.

## Funding

The authors thank the funders who made this work possible: French Ministry of Agriculture—General Directorate for Food (DGAl, grant agreement: SPA17 n°0079-E), European Funds for Regional Development (FEDER, Grand-Est), Monitoring Outbreaks for Disease surveillance in a data science context project (MOOD project, EU H2020, grant agreement ID: 874850), French Establishment for Fighting Zoonoses (ELIZ) and the Association Nationale Recherche Technologie (ANRT, grant agreement No.: 2019-1145).

## Conflict of Interest

The authors declare that the research was conducted in the absence of any commercial or financial relationships that could be construed as a potential conflict of interest.

## Publisher's Note

All claims expressed in this article are solely those of the authors and do not necessarily represent those of their affiliated organizations, or those of the publisher, the editors and the reviewers. Any product that may be evaluated in this article, or claim that may be made by its manufacturer, is not guaranteed or endorsed by the publisher.
